# Characteristics of Primary Bradyarrhythmia in Hypertrophic Cardiomyopathy: A 10-Year, Single-Center Analysis

**DOI:** 10.3390/jcdd9110370

**Published:** 2022-10-29

**Authors:** Hong-Da Zhang, Min Tang, Jing-Tao Zhang

**Affiliations:** Arrhythmia Center, State Key Laboratory of Cardiovascular Disease, Fuwai Hospital, National Center for Cardiovascular Diseases, Chinese Academy of Medical Sciences & Peking Union Medical College, Beijing 100037, China

**Keywords:** hypertrophic cardiomyopathy, bradyarrhythmia, pacemaker implantation

## Abstract

Background: Secondary bradyarrhythmias in hypertrophic cardiomyopathy (HCM) have been extensively reported. The prevalence and characteristics of primary bradyarrhythmias in HCM have yet to be investigated. Methods: We retrospectively enrolled 101 consecutive patients with HCM who were referred to the arrhythmia center from May 2010 to October 2020. The clinical features of patients with bradyarrhythmias were analyzed. Results: Twenty-nine (28.7%) patients had primary bradyarrhythmias, and six (5.9%) patients had secondary third-degree atrioventricular block (AVB). Of the 29 patients, 17 (58.6%) had sinus node dysfunction (SND), 14 (48.3%) had AVB, and two (6.9%) had both SND and AVB. The median age was 62 years old, and 69% were male. Six (20.7%) patients had left ventricular obstructive outflow tract obstruction, 15 (51.7%) had a history of syncope, and one (3.4%) had a family history of HCM. Most patients (86.2%) had New York Heart Association functional class I or II, and the median left ventricular ejection fraction was 63%. A total of 22 patients received pacemaker implantation, including 17 (77.3%) dual-chamber pacing, four (18.2%) single-chamber ventricular pacing, and one (4.5%) cardiac resynchronization therapy. Conclusions: Primary bradyarrhythmias need to be evaluated in HCM patients with arrhythmia-related symptoms. Patients with HCM might need pacemaker implantation for primary bradyarrhythmias.

## 1. Introduction

Patients with hypertrophic cardiomyopathy (HCM) often experience different kinds of arrhythmias, including both tachyarrhythmias and bradyarrhythmias [1,2]. Atrial fibrillation is the most common, and ventricular fibrillation is the most fatal arrhythmia in HCM, and they have been extensively investigated [1,2,3,4]. Symptomatic bradycardia caused by sinus node dysfunction (SND) and atrioventricular block (AVB) is relatively uncommon in HCM [2]. Most studies on bradycardia focused on secondary third-degree AVB after either ventricular septal myectomy (the Morrow procedure) or percutaneous septal alcohol or radiofrequency ablation [5]. Few studies have evaluated the primary bradyarrhythmias in HCM. This study aimed to comprehensively investigate the characteristics of primary bradyarrhythmias in patients with HCM. The prevalence and characteristics of primary bradyarrhythmias in hypertrophic cardiomyopathy (HCM) have yet to be investigated. The results show that 29 (28.7%) of the enrolled patients with HCM had primary bradyarrhythmias. We suggest that primary bradyarrhythmias be evaluated in HCM patients with arrhythmia-related symptoms.

## 2. Materials and Methods

### 2.1. Study Population

Between May 2010 and October 2020, 101 consecutive patients with HCM who had arrhythmia-related symptoms were admitted to the Arrhythmia Center, Fuwai Hospital, Beijing, for electrophysiological evaluation. The symptoms included palpitations, syncope, or presyncope. The final inclusion criteria included: (1) diagnosed with HCM; (2) primary SND or AVB. The exclusion criteria included: (1) secondary AVB due to ventricular septal myectomy or percutaneous septal alcohol ablation; (2) secondary SND or AVB due to drugs, intrinsic diseases other than cardiomyopathy, or extrinsic causes listed in the current HCM guidelines [6]. Whether the bradyarrhythmia was primary or secondary was adjudicated by three independent reviewers, two electrophysiologists from the Arrhythmia Center, and one expert from the Cardiomyopathy Center. This was conducted through a detailed chart review. This study was performed in accordance with the Declaration of Helsinki and was approved by the Institutional Review Board and ethics committee on 6 December 2021 (Approval No. 2021-1574). Informed consent was obtained from all participants.

### 2.2. Diagnosis of HCM

HCM was defined by a wall thickness ≥15 mm in one or more left ventricular myocardial segments measured by echocardiography and/or cardiovascular magnetic resonance imaging (MRI), which was not explained solely by loading conditions [1,2]. In patients with intensive physical training, hypertension, or valve diseases, the diagnosis was made by at least two experts from the Cardiomyopathy Center based on additional information, including family history, noncardiac symptoms and signs, ECG abnormalities, laboratory tests, and multimodality cardiac imaging, especially cardiac MRI. In this study, all patients were evaluated by echocardiography, and nearly half of them were also assessed by cardiac MRI. Left ventricular outflow tract obstruction was defined as an instantaneous peak Doppler left ventricular outflow tract pressure gradient of ≥30 mmHg [1,2]. A gradient ≥50 mmHg was considered hemodynamically significant [1,2].

### 2.3. Diagnosis of SND and AVB

Bradyarrhythmias included in this study were SND and AVB. Bundle branch block was not included in this study. SND included sinus bradycardia and sinus node arrest. ABV included first-degree, second-degree, and third-degree AVB. At least two electrophysiologists made the diagnosis based on ECG and Holter monitoring.

### 2.4. Follow-Up

Patients were followed up by outpatient visits or telephone calls at 3 months, 6 months, 12 months, and every year thereafter. All-cause deaths and cardiac death events were recorded. The final census date for this study was 15 December 2021.

### 2.5. Statistical Analysis

Continuous variables are expressed as mean ± standard deviation (SD) or median (interquartile range (IQR)) as appropriate. Categorical parameters are shown as ratios or percentages. The Student’s *t*-test or Mann–Whitney U test was conducted between two independent samples as appropriate for continuous data. The chi-square test was used for categorical data. A *p*-value of <0.05 was considered statistically significant. Data analyses were performed using R version 4.0.2.

## 3. Results

### 3.1. The Spectrum of Arrhythmias

**Figure 1:**
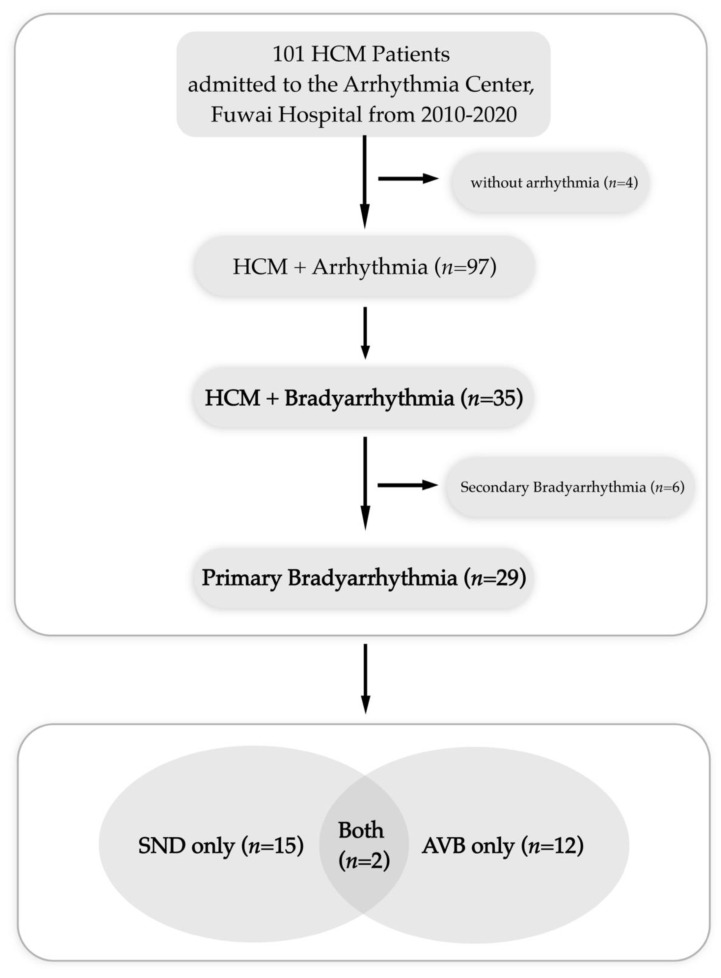
Flow chart of all patients with arrhythmias. HCM, hypertrophic cardiomyopathy; SND, sinus node dysfunction; AVB, atrioventricular block.

Figure 1 displays the spectrum of all arrhythmias. Of all 101 patients, 97 had arrhythmias, 35 had bradyarrhythmias, and 29 had primary bradyarrhythmias. Of the six patients with secondary third-degree AVB, five were due to the Morrow procedure, and one was due to percutaneous septal radiofrequency ablation. Of the 29 patients with primary bradyarrhythmias, 15 (51.7%) only had SND, 12 (41.4%) only had AVB, and two (6.9%) had both.

Of the 17 patients with SND, 12 had sinus bradycardia, six had sinus arrest, and one had both sinus bradycardia and sinus arrest. Of the 14 patients with AVB, five had first-degree AVB, seven had second-degree AVB, and five had third-degree AVB.

### 3.2. Baseline Characteristics of Patients with Bradyarrhythmias

The demographic and clinical features of all patients with primary bradyarrhythmias are shown in Table 1. In the overall cohort, 20 (69%) patients were male, and the median (IQR) age at admission was 62 (32.5) years old. Fifteen (51.7%) patients had a history of syncope, and one (3.4%) had a family history of HCM. Hypertension was the most frequent (48.3%) comorbid disease. Cardiac MRI was performed in 12 patients, and late gadolinium enhancement was detected in 10 of these patients.

The echocardiography parameters are displayed in Table 2. The multisegment (48.3%) and interventricular septum (37.9%) comprised the majority of hypertrophic types. Six (20.7%) patients had left ventricular obstructive outflow tract obstruction. The mean maximum left ventricular wall thickness was 20 ± 4.3 mm. Most patients had a normal systolic function with a median (IQR) left ventricular ejection fraction of 63% (10.0).

Comparisons of all the baseline characteristics between the two groups are shown in Table 3. More patients with SND had left ventricular obstructive outflow tract obstruction than those with AVB (*p* = 0.013). Other clinical features were comparable between the two groups.

The baseline characteristics were also compared between male and female patients (Table 4). Male patients had greater levels of weight, height, and body surface area than female patients, as expected. More male patients had coronary artery disease than female patients (*p* = 0.042). Male patients seemed to have greater maximum left ventricular wall thickness than female patients with a borderline significant trend (*p* = 0.067). Other clinical features were comparable between the two groups.

### 3.3. Treatment and Outcomes

A total of 22 patients received pacemaker implantation, including 17 (77.3%) dual-chamber pacing, four (18.2%) single-chamber ventricular pacing, and one (4.5%) cardiac resynchronization therapy. The reasons for pacemaker implantation were AVB in 12 patients, SND in 10 patients, and none for improving HCM-related symptoms. At discharge, 20 (69.3%) patients received beta-blockers, and three (10.3%) patients received non-dihydropyridine calcium channel blockers to improve HCM-related symptoms (Table 1). There were no death events during follow-up.

## 4. Discussion

HCM is one of the leading causes of sudden cardiac death in youths and athletes. Arrhythmias may cause palpitations, syncope, or presyncope in patients with HCM. Syncope could be caused by ventricular tachycardia, supraventricular tachycardia, or bradyarrhythmias, including SND and AVB. Although ventricular fibrillation is the most commonly recorded fatal arrhythmic event, asystole and AVB have been reported [2]. Primary bradyarrhythmias were believed to be uncommon in patients with HCM, and hence are understudied. This study aimed to comprehensively analyze different types of primary bradyarrhythmias in a tertiary-based HCM population in China. The results indicate that more than a quarter (29/101) of the patients with arrhythmia-related symptoms had primary bradyarrhythmias. The prevalences of SND and AVB were similar.

Our study shows that some patients with symptomatic SND and AVB required pacemaker implantation. There are many studies on pacemaker implantation in patients with HCM, in which the indications are improving the HCM-related symptoms or secondary third-degree AVB. There are case reports on patients with HCM and primary third-degree AVB [7,8,9]. In a study by Barriales-Villa and colleagues [10], of 48 patients with HCM who received pacemaker implantation, 20 had SND (16 were primary, and four were secondary), and 28 had AVB (20 were primary, and eight were secondary). Another study [11] included 70 patients with HCM who received a pacemaker implantation, and 22 (31%) of them were due to bradyarrhythmias. Of the 22 patients, 12 were AVB, six were SND, and four were atrial fibrillation with a slow ventricular rate. However, the authors did not mention how many patients had primary or secondary bradyarrhythmias.

Higuchi et al. reported that 96 (23.2) of the 414 patients with HCM had first-degree AVB [12]. In the present study, the figure for first-degree AVB was 5%, which might be greater if we expanded the population to the general HCM population.

The presence of AVB might be related to particular genetic subtypes in younger patients. In this study, we could not obtain genotype information. However, patients with AVB in this study were older, with a median age of 56 years.

## 5. Limitations

This study had several limitations. Firstly, there was patient selection bias because this was a retrospective analysis from a single tertiary arrhythmia center. The population enrolled did not represent the general HCM population. Secondly, the sample size was small, although this was a 10-year analysis. Thirdly, genotype information was not available in the present study, which might be important for classifying the etiology of HCM. Lastly, in this study, patients had a mean age of 62 years, and we could not rule out the association between age and bradyarrhythmias. Further multicenter studies of primary bradyarrhythmias in the general HCM population are needed.

## 6. Conclusions

Primary bradyarrhythmias need to be evaluated in HCM patients with arrhythmia-related symptoms. Patients with HCM might need a pacemaker implantation for primary bradyarrhythmias.

## Figures and Tables

**Table 1:** Demographic and clinical characteristics.

Parameters	Overall (*n* = 29)	SND (*n* = 17)	AVB (*n* = 14)
Male gender, *n* (%)	20 (69.0)	11 (64.7)	11 (78.6)
Weight, kg	67 ± 11.8	66 ± 12.6	67 ± 11.7
Height, cm	168 ± 6.6	168 ± 6.9	169 ± 6.3
BSA, m^2^	1.75 ± 0.17	1.74 ± 0.17	1.76 ± 0.16
BMI, kg/m^2^	23.5 ± 3.42	23.4 ± 3.68	23.3 ± 3.39
Age at admission, y	62 (32.5)	65 (18.5)	56 (32.5)
Age at arrhythmia symptom onset, y	54 (30.0)	55 (28.0)	50 (27.0)
History of syncope, *n* (%)	15 (51.7)	9 (52.9)	7 (50)
Family history of HCM, *n* (%)	1 (3.4)	0 (0)	1 (7.1)
Comorbidities, *n* (%)	19 (65.5)	12 (70.6)	8 (57.1)
HTN, *n* (%)	14 (48.3)	9 (52.9)	6 (42.9)
CAD, *n* (%)	7 (24.1)	4 (23.5)	3 (21.4)
DM, *n* (%)	3 (10.3)	1 (5.9)	2 (14.3)
CHD, *n* (%)	0 (0)	0 (0)	0 (0)
VHD, *n* (%)	0 (0)	0 (0)	0 (0)
PH, *n* (%)	3 (10.3)	1 (5.9)	3 (21.4)
History of Stroke, *n* (%)	1 (3.4)	1 (5.9)	0 (0)
NYHA-FC, *n* (%)			
I/II	25 (86.2)	15 (88.2)	11 (78.6)
III/IV	4 (13.8)	2 (11.8)	3 (21.4)
HCM symptom-improving therapy at discharge			
Beta-blockers	20 (69.0)	12 (70.6)	8 (51.7)
Non-DHP CCB	3 (10.3)	3 (17.6)	0 (0)

SND, sinus node dysfunction; AVB, atrioventricular block; BSA, body surface area; BMI, body mass index; HCM, hypertrophic cardiomyopathy; HTN, hypertension; CAD, coronary artery disease; DM, diabetes mellitus; CHD, congenital heart disease; VHD, valvular heart disease; PH, pulmonary hypertension; NYHA-FC, New York Heart Association functional class; Non-DHP CCB, Non-dihydropyridine calcium channel blocker. Values are expressed as *n* (%), ratio, mean ± SD, or median (interquartile range).

**Table 2:** Echocardiography parameters.

Parameters	Overall (*n* = 29)	SND (*n* = 17)	AVB (*n* = 14)
HCM types			
IVS hypertrophy	11 (37.9)	6 (35.3)	6 (42.9)
Apex hypertrophy	4 (13.8)	4 (23.5)	0 (0)
Multisegment hypertrophy	14 (48.3)	7 (41.2)	8 (57.1)
LVOT obstruction, *n*(%)	6 (20.7)	6 (35.3)	0 (0)
Ejection fraction, %	63 (10.0)	65 (11.5)	60 (13.5)
LA dimension (AP), mm	41 (7.0)	41 (9.0)	40 (4.8)
LVEDD, mm	47 ± 6.8	46 ± 6.2	49 ± 8.9
Maximum LV thickness, mm	20 ± 4.3	19 ± 4.6	21 ± 3.7
Mitral regurgitation ≥ moderate, *n* (%)	2 (6.9)	2 (9.5)	1 (6.3)

SND, sinus node dysfunction; AVB, atrioventricular block; HCM, hypertrophic cardiomyopathy; IVS, interventricular septum; LVOT, left ventricular outflow tract; LA, left atria; AP, anteroposterior; LVEDD, left ventricular end-diastolic dimension; LV, left ventricle. Values are expressed as *n* (%), ratio, mean ± SD, or median (interquartile range).

**Table 3:** Comparisons between patients with SND and patients with AVB.

Parameters	SND Only (*n* = 15)	AVB Only (*n* = 12)	*p* Value
Male gender, *n*(%)	9 (60.0)	9 (75.0)	0.411
Weight, kg	67 ± 12.3	68 ± 10.9	0.817
Height, cm	168 ± 7.1	169 ± 6.6	0.574
BSA, m^2^	1.75 ± 0.17	1.77 ± 0.16	0.693
BMI, kg/m^2^	23.8 ± 3.53	23.7 ± 3.15	0.965
Age at admission, y	66 (13.0)	56 (39.0)	0.252
Age at arrhythmia symptom onset, y	62 (26.0)	50 (29.0)	0.704
History of syncope, *n*(%)	8 (53.3)	6 (50.0)	0.863
Family history of HCM, *n*(%)	0 (0)	1 (8.3)	0.255
Comorbidities, *n*(%)			
HTN, *n*(%)	8 (53.3)	5 (41.7)	0.547
CAD, *n*(%)	4 (26.7)	3 (25.0)	0.922
DM, *n*(%)	1 (6.7)	2 (16.7)	0.411
CHD, *n*(%)	0 (0)	0 (0)	N/A
VHD, *n*(%)	0 (0)	0 (0)	N/A
PH, *n*(%)	0 (0)	2 (16.7)	0.100
History of Stroke, *n*(%)	1 (6.7)	0 (0)	0.362
NYHA-FC, *n*(%)			0.268
I/II	14 (93.3)	10 (83.3)	
III/IV	1 (6.7)	2 (16.7)	
HCM symptom improving therapy			
Beta-blockers	12 (80.0)	8 (66.7)	0.432
Non-DHP-CCB	3 (20.0)	0 (0)	0.100
Echocardiography parameters			
HCM types			0.150
IVS hypertrophy	5 (33.3)	5 (41.7)	
Apex hypertrophy	4 (26.7)	0 (0)	
Multi-segment hypertrophy	6 (40.0)	7 (58.3)	
LVOT Obstruction, *n*(%)	6 (40.0)	0 (0)	0.013
Ejection fraction, %	65 (8.0)	61 (10.0)	0.252
LA dimension (AP), mm	41 (11.0)	40 (6.3)	0.696
LVEDD, mm	46 ± 3.8	50 ± 7.2	0.126
Max LV thickness, mm	19 ± 4.6	21 ± 3.9	0.247
Mitral regurgitation ≥moderate, *n*(%)	2 (10.5)	0 (0)	0.482

SND, sinus node dysfunction; AVB, atrioventricular block; BSA, body surface area; BMI, body mass index; HCM, hypertrophic cardiomyopathy; IVS, interventricular septum; LVOT, left ventricular outflow tract; LA, left atrial; AP, anteroposterior; LVEDD, left ventricular end-diastolic dimension; LV, left ventricle; HTN, hypertension; CAD, coronary artery disease; DM, diabetes mellitus; CHD, congenital heart disease; VHD, valvular heart disease; PH, pulmonary hypertension; NYHA-FC, New York Heart Association functional class; Non-DHP CCB, Non-dihydropyridine calcium channel blocker; N/A, not applicable. Values are expressed as *n* (%), ratio, mean ± SD, or median (interquartile range). Comparisons between the two groups: student *t*-test or Mann-Whiney U test for continuous data, and chi-square test for categorical data.

**Table 4:** Comparisons between male and female patients.

Parameters	Male (*n* = 20)	Female (*n* = 9)	*p* Value
Weight, kg	70 ± 11.9	61 ± 9.0	0.048
Height, cm	171 ± 5.0	162 ± 5.2	<0.001
BSA, m^2^	1.81 ± 1.15	1.62 ± 0.12	0.002
BMI, kg/m^2^	23.7 ± 3.63	23.1 ± 3.03	0.639
Age at admission, y	59 ± 20.5	57 ± 13.2	0.833
Age at arrhythmia symptom onset, y	55 ± 18.7	47 ± 13.5	0.256
History of syncope, *n*(%)	11 (55.0)	4 (44.4)	0.599
Family history of HCM, *n*(%)	0 (0)	1 (11.1)	0.129
Comorbidities, *n*(%)			
HTN, *n*(%)	11 (55.0)	3 (33.3)	0.280
CAD, *n*(%)	7 (35.0)	0 (0)	0.042
DM, *n*(%)	2 (10.0)	1 (11.1)	0.928
CHD, *n*(%)	0 (0)	0 (0)	N/A
VHD, *n*(%)	0 (0)	0 (0)	N/A
PH, *n*(%)	3 (15.0)	0 (0)	0.220
History of Stroke, *n*(%)	0 (0)	1 (11.1)	0.129
NYHA-FC, *n*(%)			0.164
I/II	18 (90.0)	7 (77.8)	
III/IV	2 (10.0)	2 (22.2)	
HCM symptom improving therapy			
Beta-blockers	13 (65.0)	7 (77.8)	0.491
Non-DHP-CCB	2 (10.0)	1 (11.1)	0.928
Echocardiography parameters			
HCM types			0.491
IVS hypertrophy	7 (35.0)	4 (44.4)	
Apex hypertrophy	2 (10.0)	2 (22.2)	
Multi-segment hypertrophy	11 (55.0)	3 (33.3)	
LVOT Obstruction, *n*(%)	4 (20.0)	2 (22.2)	0.891
Ejection fraction, %	64 (10.7)	62 (11.5)	0.321
LA dimension (AP), mm	41 ± 7.3	39 ± 11.1	0.538
LVEDD, mm	46 ± 7.6	49 ± 4.3	0.280
Max LV thickness, mm	21 ± 4.4	18 ± 3.4	0.067
Mitral regurgitation ≥moderate, *n*(%)	2 (10.0)	0 (0)	0.587

BSA, body surface area; BMI, body mass index; HCM, hypertrophic cardiomyopathy; IVS, interventricular septum; LVOT, left ventricular outflow tract; LA, left atrial; AP, anteroposterior; LVEDD, left ventricular end-diastolic dimension; LV, left ventricle; HTN, hypertension; CAD, coronary artery disease; DM, diabetes mellitus; CHD, congenital heart disease; VHD, valvular heart disease; PH, pulmonary hypertension; NYHA-FC, New York Heart Association functional class; Non-DHP CCB, Non-dihydropyridine calcium channel blocker; N/A, not applicable. Values are expressed as *n* (%), ratio, mean ± SD, or median (interquartile range). Comparisons between the two groups: student *t*-test or Mann-Whiney U test for continuous data, and chi-square test for categorical data.

## Data Availability

Research data is confidential. Data sharing requests are required to meet the policies of the hospital and the funder.
